# Polymeric Engineering of Nanoparticles for Highly Efficient Multifunctional Drug Delivery Systems

**DOI:** 10.1038/s41598-019-39107-3

**Published:** 2019-02-25

**Authors:** Beatrice Fortuni, Tomoko Inose, Monica Ricci, Yasuhiko Fujita, Indra Van Zundert, Akito Masuhara, Eduard Fron, Hideaki Mizuno, Loredana Latterini, Susana Rocha, Hiroshi Uji-i

**Affiliations:** 1 KU Leuven, department of Chemistry, Celestijnenlaan 200G-F, Heverlee, 3001 Belgium; 2 RIES Hokkaido University, Research Institute for Electronic Science, N20W10, Kita-Ward Sapporo, 0010020 Japan; 3 Toray Research Center, Inc., 3-3-7, Sonoyama, Otsu, Shiga 520-8567 Japan; 4 Yamagata University, department of Engineering, Yonezawa, Yamagata 992-8510 Japan; 5 University of Perugia, department of Chemistry, Biology and Biotechnology, via Elce di sotto 8, Perugia, Italy

## Abstract

Most targeting strategies of anticancer drug delivery systems (DDSs) rely on the surface functionalization of nanocarriers with specific ligands, which trigger the internalization in cancer cells via receptor-mediated endocytosis. The endocytosis implies the entrapment of DDSs in acidic vesicles (endosomes and lysosomes) and their eventual ejection by exocytosis. This process, intrinsic to eukaryotic cells, is one of the main drawbacks of DDSs because it reduces the drug bioavailability in the intracellular environment. The escape of DDSs from the acidic vesicles is, therefore, crucial to enhance the therapeutic performance at low drug dose. To this end, we developed a multifunctionalized DDS that combines high specificity towards cancer cells with endosomal escape capabilities. Doxorubicin-loaded mesoporous silica nanoparticles were functionalized with polyethylenimine, a polymer commonly used to induce endosomal rupture, and hyaluronic acid, which binds to CD44 receptors, overexpressed in cancer cells. We show irrefutable proof that the developed DDS can escape the endosomal pathway upon polymeric functionalization. Interestingly, the combination of the two polymers resulted in higher endosomal escape efficiency than the polyethylenimine coating alone. Hyaluronic acid additionally provides the system with cancer targeting capability and enzymatically controlled drug release. Thanks to this multifunctionality, the engineered DDS had cytotoxicity comparable to the pure drug whilst displaying high specificity towards cancer cells. The polymeric engineering here developed enhances the performance of DDS at low drug dose, holding great potential for anticancer therapeutic applications.

## Introduction

Over the last few decades, the engineering of nanoparticles has given rise to significant breakthroughs towards the employment of nanomaterials in biomedical applications, such as cancer therapy, (bio-) chemical sensing, and bio-imaging^1–5^. In particular, mesoporous silica nanoparticles (MSNPs) have been widely applied as promising anticancer drug nanocarriers thanks to their biocompatibility, high loading capacity, chemical stability and straightforward synthesis/surface functionalization^6–8^. Unlike some other nanocarriers, MSNPs have not been translocated into the clinical stage yet^9^. However, the reasonable biocompatibility accomplished *in vivo* is extremely promising for a proximate Food and Drug Administration (FDA-) approval^10^.

To promote the specific internalization of nanoparticles to certain cancer cells (cancer targeting), many strategies have been developed so far. These methods are mainly based on the employment of specific ligands, which can bind to receptors overexpressed in tumor cells and trigger particle internalization via endocytosis^11–14^. In this context, hyaluronic acid (HA) has gained increasing attention as targeting ligand due to its high affinity with CD44, a glycoprotein receptor overexpressed in many solid tumor cells (*e.g*. lung, breast, pancreatic, renal tumor), in metastasis, as well as in cancer stem cells^15^. As being one of the main constituents of the extracellular matrix, HA exhibits high biocompatibility, which has enabled its FDA-approval for medical and cosmetic use^16–18^. The harmlessness of HA, allied with its effective targeting capability, encouraged its employment to selectively internalize HA-functionalized materials (HA-materials) in CD44-overexpressing cancer cells via receptor-mediated endocytosis^19–26^.

In spite of the well-achieved cell-specific internalization, the control of the particle fate after overpassing the plasma membrane remains challenging, and existing strategies are still limited. In eukaryotic cells, external materials (such as nutrients, protein and lipids, as well as nanoparticles), taken up via endocytosis, are normally sorted out in endocytic vesicles (endosomes and lysosomes) and can eventually be ejected to the extracellular matrix via exocytosis^27^. Previous reports have shown that non-coated MSNPs co-localize with the endo-/lysosomes in the early stage of incubation^28–31^, and are quickly exocytosed, following this pathway^32^. Similarly, HA-coated MSNPs are internalized via CD44-mediated endocytosis and are subjected to same endocytic system, ending up in the acidic cellular compartments within few hours of incubation^23,33^, and being ejected via exocytosis within 48 h^34^. The endo-/exocytosis process represents one of the main hindrances of the DDSs in light of the limited cargo release in the intracellular environment. The low lysosomal pH (4.5–5.5 for normal cells and 3.5–5 for cancer cells) and the strong enzymatic activity might lead to drug degradation, possibly inhibiting its pharmaceutical activity^35^. The therapeutic efficiency can be further decreased by the fast exocytosis of the nanocarriers^36^. As the drug release normally occurs by slow diffusion, the DDS can be exocytosed to the extracellular matrix before releasing all its cargo, contributing to the low therapeutic performance (forcing the use of higher drug dose), as well as to chemotherapy side effects. Despite the major consequences in terms of therapy efficiency, the intracellular route of nanocarriers is often neglected in the development of novel DDS, and strategies to enable the escape from this endocytic route are very limited. To this end, the employment of cationic polymers, in particular polyethylenimine (PEI), is a promising strategy, as it is non-immunogenic and easier to scale up, compared to other agents, such as viral proteins and synthetic fusogenic peptides^37,38^. PEI is already widely used in DNA transfection for promoting the release of genetic material from the acidic vesicles and, thus, facilitating the incoming to the nucleus^39,40^. The use of DNA-PEI polyplexes, instead of pure DNA, was demonstrated to improve the gene expression efficiency up to 100-fold^40,41^. This enhanced gene expression can be associated to the so-called “proton sponge effect” of PEI^42^. Most specifically, thanks to the protonation of tertiary amines, PEI exhibits high buffering capability at low pH, promoting an influx of protons inside the acidic cellular compartments via ATPase proton pumps and the consequent rupture of the organelle membrane due to an osmotic imbalance. The proton sponge effect of PEI is a generally accepted hypothesis in literature, however, it is important to mention that this concept is still heavily debated^43^.

Since the action mechanism of most anticancer drugs, *e.g*. doxorubicin (Dox), is based on its intercalation into DNA and complex formation with DNA-associated enzymes^44^, the same approach can be used to enhance the nuclear delivery of anticancer drugs. The main hindrance for the application of PEI on DDSs is its cytotoxicity, which can be, howbeit, drastically reduced by using a low molecular weight (0.5–5 kDa)^45,46^. So far, PEI has been used to functionalize MSNPs for the successful delivery of either siRNA/DNA or siRNA/doxorubicin to HEPA-1 and KB-V1 cells, respectively^45,47^. In these studies, the endosomolytic activity of the PEI layer was assumed but not verified. On the other hand, Yanes *et al*. demonstrated that the addition of a PEI layer can slow down the exocytosis rate of MSNPs, although no investigation on the intracellular distribution of the nanoparticles was performed^48^. To the best of our knowledge, a study on the intracellular sorting of PEI-coated nanocarriers, which provides an evidence of their endosomal escape, has never been reported.

**Figure 1:**
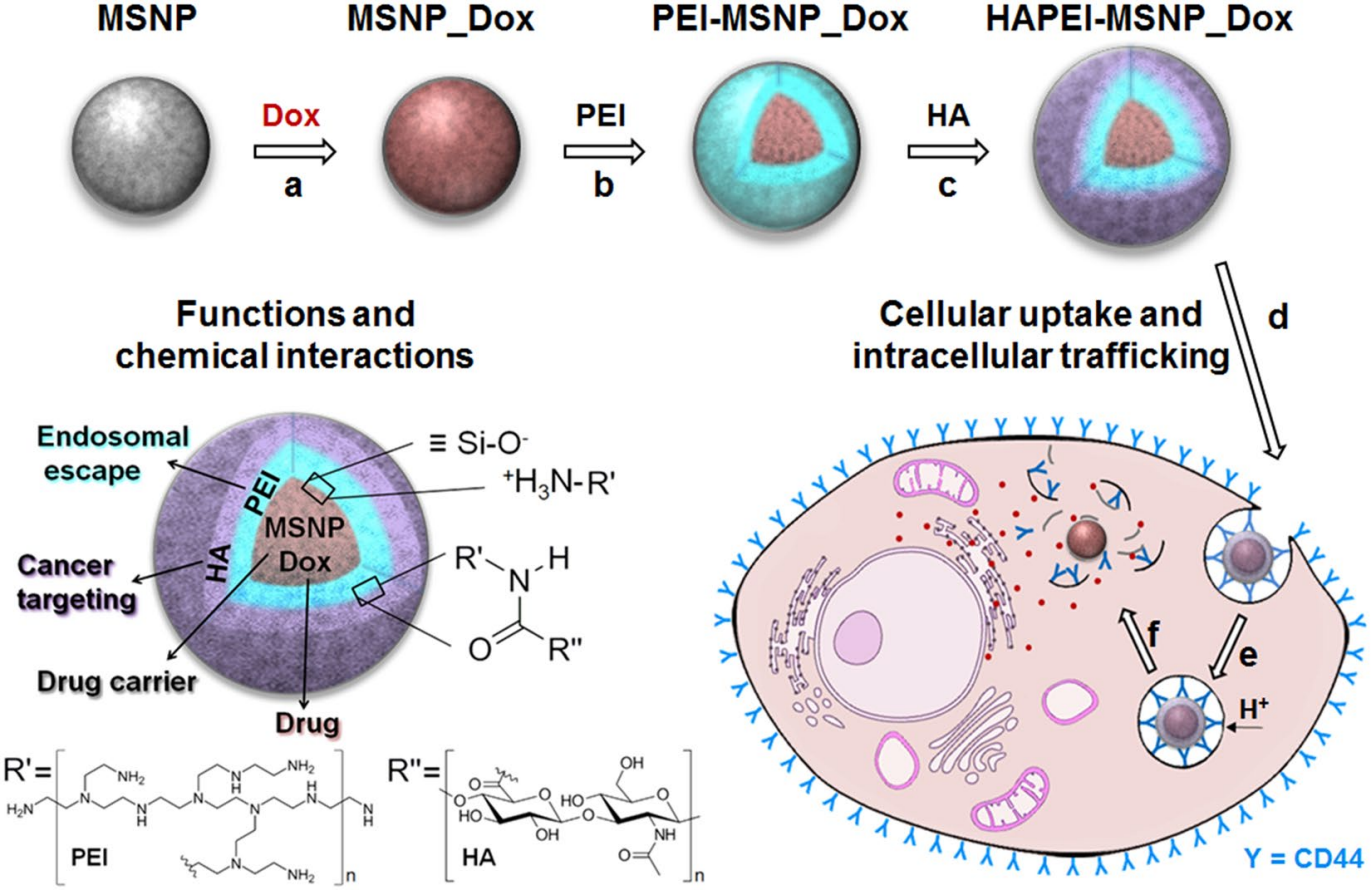
Multifunctional drug delivery system based on MSNPs: particle synthesis and cellular trafficking. (**a**–**c**) Preparation of HAPEI-MSNP_Dox: (**a**) encapsulation of doxorubicin (Dox) inside mesoporous silica nanoparticles (MSNP_Dox); (b) coating with polyethylenimine (PEI) layer (PEI-MSNP_Dox); (**c**) surface grafting with hyaluronic acid (HA) (HAPEI-MSNP_Dox). (**d**–**f**) Cellular uptake and intracellular trafficking: (**d**) particle interaction with the plasma membrane via CD44-HA site-specific binding; (**e**) HAPEI-MSNP_Dox uptake via receptor-mediated endocytosis and wrapping in endosomes; (**f**) rupture of the endosomal membrane upon proton sponge effect of PEI and drug release into the cytoplasm. A schematic illustration representing functions and chemical interactions of each component is reported at left-bottom of the figure.

In this manuscript, we propose a facile method to functionalize mesoporous silica nanoparticles with a polymeric bilayer, which simultaneously combines the active targeting action of HA and PEI-mediated endosomal escape (Fig. 1). For therapeutic applications, any anticancer drug can be loaded in the particles. Here, we use Dox-loaded MSNPs and show that the combination of active targeting, endosomal escape and controlled drug release results in high therapy efficiency. The method presented paves the way for the development of the next generation highly efficient DDSs.

## Results and Discussion

### Preparation and characterization of multifunctional MSNPs

**Figure 2:**
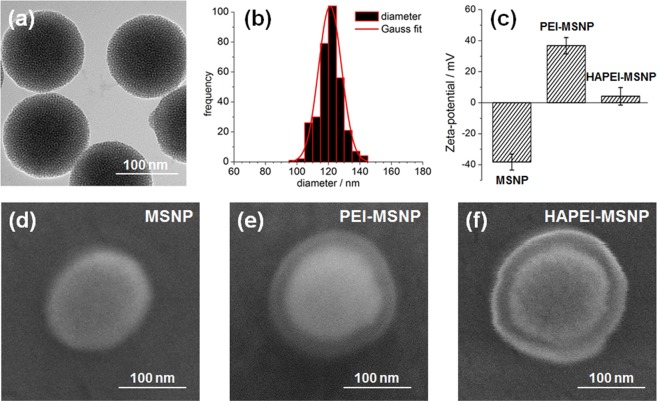
Characterization of MSNPs and their surface modifications. (**a**) TEM image of bare MSNPs. (**b**) Size distribution of the MSNPs (Gauss distribution in red fitting). (**c**) Zeta potential measurements of MSNPs, PEI-MSNPs and HAPEI-MSNPs. (**d**–**f**) FE-SEM images of MSNPs, PEI-MSNPs and HAPEI-MSNPs, respectively.

Due to their popularity as highly stable, low-cost and reasonably biocompatible nanocarriers, mesoporous silica nanoparticles (MSNPs) were chosen as model of nanoparticle for the application of the polymeric coating here proposed^10,49^. MSNPs were synthetized using the biphase stratification method developed by Shen *et al*., that yields particles with a pore size of ~2.8 nm^50^. Transmission electron microscopy (TEM) images of uncoated MSNPs clearly show a uniform mesoporous frame (Fig. 2a). The particles exhibit size and shape homogeneity, with no observable aggregates. As depicted in Fig. 2b, the mean diameter was estimated to be 120 nm. After the synthesis, MSNPs were loaded with rhodamine B (RhodB) or fluorescein isothiocyanate (FITC) for monitoring cellular uptake/trafficking, and with Dox for testing the drug release and the therapeutic effect in cancer mammalian cells. The successful loading of dye/drug inside the pores was verified by fluorescence microscopy (Supplementary Fig. S1a–c).

In order to provide the DDS with endosomal escape capability, MSNPs were coated with PEI (~1.3 kDa). Besides their biocompatibility and high loading capability, MSNPs offer a negatively charged surface which facilitates any kind of electrostatic interaction-based functionalization. At physiological pH, primary and secondary amines of PEI are protonated (pK_a_ 8–10, depending on the molecular weight of the polymer)^51,52^, whereas ~50% of hydroxyl groups on a silica surface are deprotonated (pK_a_ ≈ 6.8)^53^. This ionization percentage enables the formation of a PEI shell on the MSNP surface via electrostatic interaction. The presence of the PEI layer on MSNPs was demonstrated by the drastic change in the zeta potential of the particles after the coating (from −38.2 mV to +37.7 mV, Fig. 2c).

Thanks to the abundance of amino groups, the presence of PEI on the surface of MSNPs allowed for a straightforward binding of the targeting agent, HA, without any extra chemical modification. The carboxylic group of HA was covalently linked to the amino group of PEI via carbodiimide crosslinking reaction^23^. The decrease of the electrokinetic potential from +37.7 to +4.2 mV after HA grafting onto the PEI coating indicates the successful functionalization of the particles with HA (Fig. 2c). Considering such a change of the electrokinetic potential upon HA grafting, an effect on the charge-based PEI coating cannot be excluded. On the other hand, no attachment of HA would occur without the presence of a PEI layer on the surface, suggesting that the electrostatic interactions between PEI and the silanol groups endure the HA grafting process.

The presence of the polymeric layers was confirmed using high resolution field-emission scanning electron microscopy (FE-SEM). Representative images of bare MSNPS, MSNPS coated with PEI (PEI-MSNPs) and MSNPs functionalized with a bilayer of PEI and HA (HAPEI-MSNPs) are shown in Fig. 2d–f, respectively. While the PEI layer is barely visible in the FE-SEM images of PEI-MSNPs (Fig. 2e), after conjugation with HA, the edge contrast increases, enabling an easier visualization of the polymeric layers in Fig. 2f. It is important to note that during image acquisition the coating collapsed and detached from the silica surface as a consequence of exposure to high accelerating voltages (30 kV). Therefore, the thickness of the layers visible in Fig. 2e,f does not correspond to the exact thickness of the shells. The halo displayed in Fig. 2e,f was never observed for bare MSNPs (additional FE-SEM images of bare MSNPs and HAPEI-MSNPs in Supplementary Fig. S2).

### Cellular uptake: HA-mediated active targeting

In order to evaluate the targeting efficiency and the cell specificity of the external functionalization with HA, we monitored the cellular uptake of the different particles into two mammalian cell lines. Most specifically, RhodB-loaded MSNPs with different coatings were added to A549 cells (CD44-overexpressing cells, derived from human lung carcinoma)^54^ and NIH3T3 (mouse embryonic fibroblasts, lowly expressing CD44 receptors, defined as CD44-negative or CD44-inactive cells)^55^.

**Figure 3:**
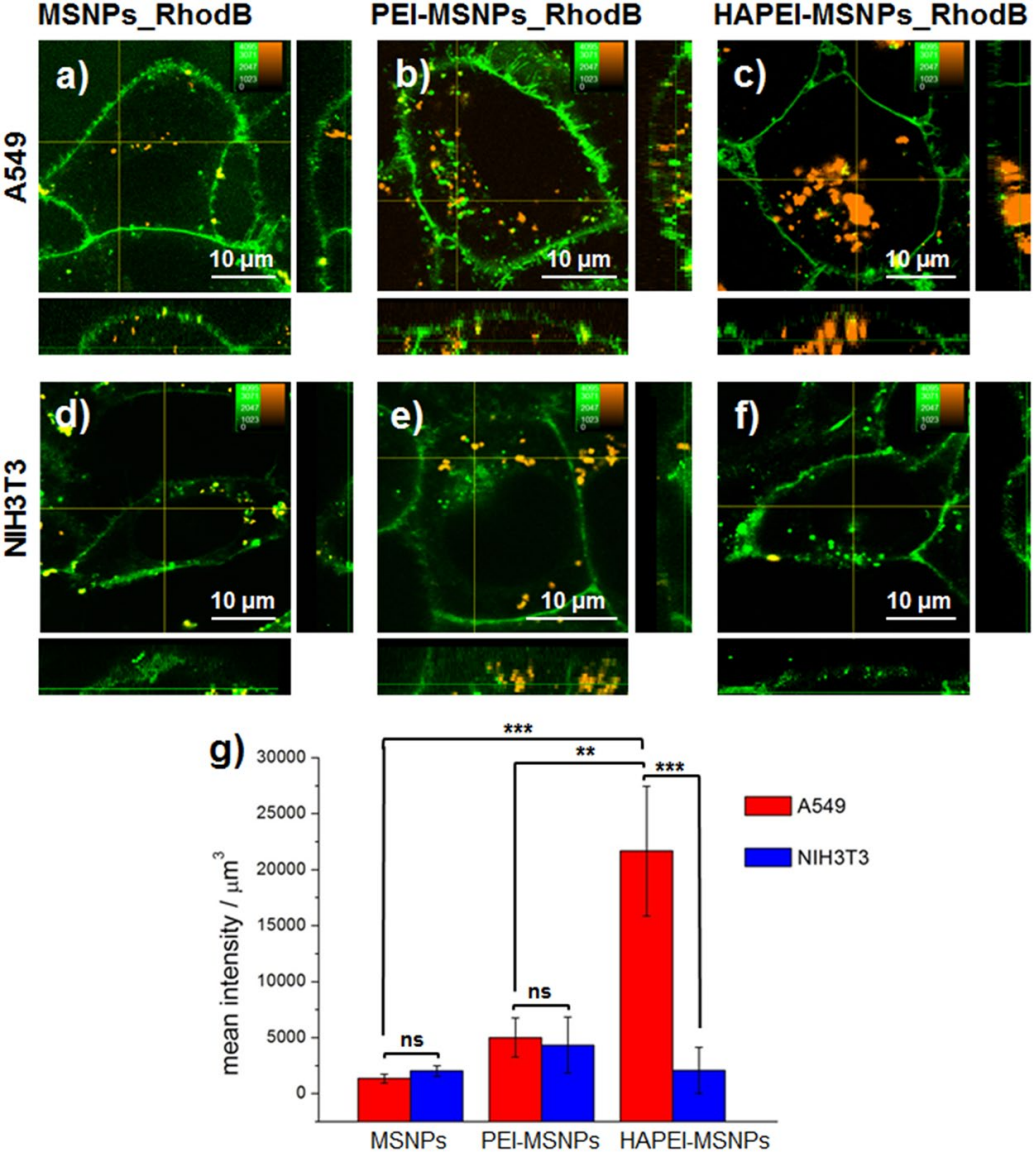
Influence of surface modification on the cellular uptake of MSNPs. (**a**–**f**) Fluorescence images of A549 and NIH3T3 cells after incubation with MSNPs_RhodB, PEI-MSNPs_RhodB and HAPEI-MSNPs_RhodB for 3 h. RhodB-loaded particles are shown in orange; DiO-stained plasma membrane is colored in green. The central panel displays an xy-plane within the cells, while the right and bottom panels show the yz and xz projections, respectively. (**g**) Mean intensity of the RhodB signal per μm^3^ of cell (n = 4 for each condition), error bars indicate ± SD, with ns = (p > 0.05), *(p < 0.05), **(p < 0.01) and ***(*p < *0.001).

Figure 3 shows typical fluorescence images of both cell lines after 3 h of incubation with the different nanoparticles, with no coating (MSNPs), only a PEI layer (PEI-MSNPs) or functionalized with both HA and PEI (HAPEI-MSNPs). In order to quantify the cellular uptake, the plasma membrane was stained with a membrane-incorporating fluorescent dye (DiO, shown in green in Fig. 3). While there was a minimal amount of bare MSNPs detected inside the cells (Fig. 3a,d,g), PEI-MSNPs show a 2-fold increase in cellular uptake, independently of the cell line (Fig. 3b,e,g). This is in agreement with previously published results^45^ and is linked to the positive charge of PEI, which boosts electrostatic interactions with the electronegative plasma membrane and facilitates particle internalization. The addition of HA minimizes the surface charge of the PEI coated nanoparticles and reduces this effect. Consequently, in NIH3T3 cells, the uptake of HAPEI-MSNPs is similar to that of bare MSNPs (Fig. 3f). Remarkably, incubation of A549 cells with HAPEI-MSNPs results in a 10-fold increase on the amount particles detected inside the cell (compared with bare MSNPs, Fig. 3c and g). The drastic discrepancy in HAPEI-MSNP uptake rate between NIH3T3 (Fig. 3f) and A549 cell lines (Fig. 3c) proves that the HA functionalization provides our DDS (HAPEI-MSNP) with high specificity towards CD44-overexpressing cancer cells.

### Intracellular trafficking: PEI-induced endosomal rupture

**Figure 4:**
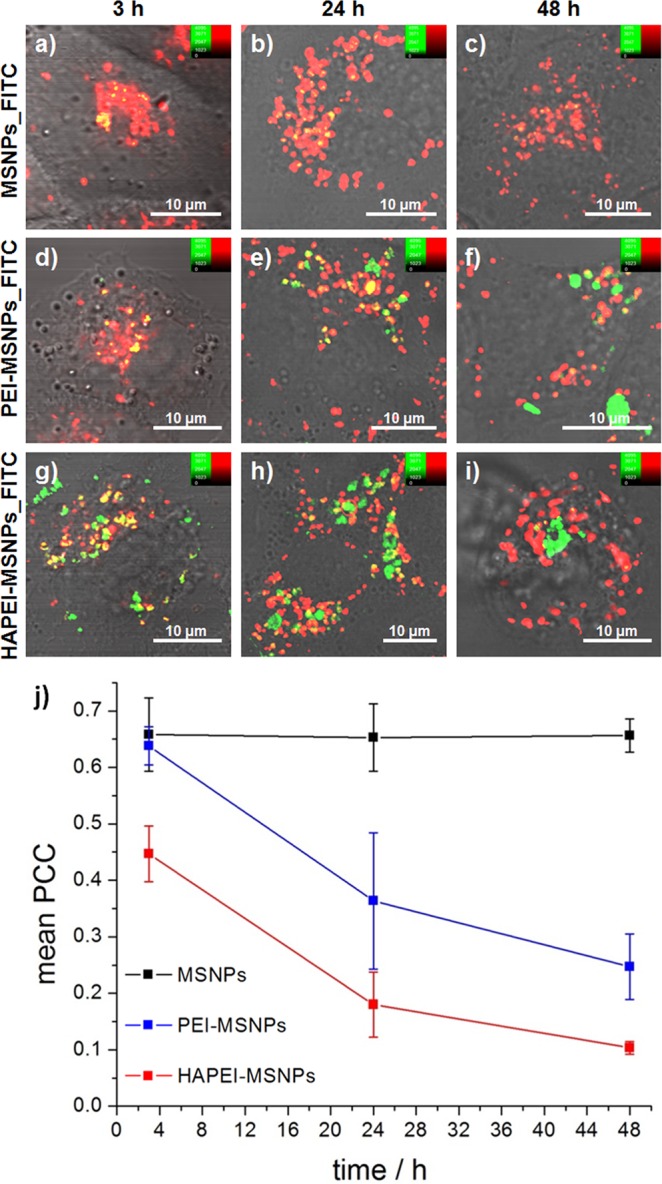
Influence of surface modification on the intracellular trafficking of MSNPs at different time points. (**a**–**i**) Fluorescence images of A549 cells incubated with MSNPs_FITC (**a**–**c**), PEI-MSNPs_FITC (**d**–**f**) and HAPEI-MSNPs_FITC (**g**–**i**) after 3, 24 and 48 h of incubation. The lysosomes were stained using LysoTracker Red®. Green channel (FITC-loaded particles), red channel (LysoTracker Red-stained endo-/lysosomes) and DIC merged images are shown. (**j**) Co-localization coefficient between the fluorescence signal of FITC-loaded nanoparticles and the LysoTracker Red (PCC ± SD plot over time, n = 5). PCC analysis was performed by using MATLAB software.

Previous reports have shown that bare and HA-functionalized MSNPs traffic through the endocytic pathway, ending up into lysosomes and, eventually, being exocytosed^23,28,30,31,33^. In order to evaluate the effect of both PEI coating alone and its combination with HA on the endosomal trafficking, A549 cells were incubated with FITC-loaded particles for 3, 24 and 48 h. It is important to mention that after 3 h of incubation, the medium was refreshed to discard the excess of particles, preventing further internalization. After the incubation period, lysosomes were stained using LysoTracker Red®, a fluorophore linked to a weak base that is only partially protonated at neutral pH and is fluorescent only in acidic environments. Cells were imaged by fluorescence microscopy and the co-localization between the fluorescence signal of FITC-loaded nanoparticles and LysoTracker Red® was determined using the Pearson’s correlation coefficient (PCC)^56^ analysis (PCC threshold values of the current study are reported in SI, Supplementary Fig. S3). Figure 4 displays representative images of A549 cells incubated with MSNPs with different coatings, after 3, 24 and 48 h. The particles are shown in green while the acidic compartments are presented in red. As a consequence, MSNPs trapped in endo- or lysosomes are displayed in yellow.

After 3 h, MSNPs without any additional surface functionalization co-localized with the endo-/lysosomes (Fig. 4a). Even after 48 h, all the particles detected inside A549 cells were co-localized with acidic compartments, indicating that none of the bare MSNPs taken up by the cell was able to escape the endocytic pathway (Fig. 4c). Accordingly, the calculated PCC is constant over time (black line in Fig. 4j). Note that, since the internalization rate of MSNPs is relatively low comparted to that of HAPEI- and PEI-MSNPs, in order to get an appropriate comparison study of the intracellular distribution, A549 cells with relatively higher MSNPs uptake were selected to perform confocal imaging and subsequent PCC analysis.

Within a time span of 3 h, the PEI coating does not induce a clear effect on the intracellular fate of the nanoparticles, with bare MSNPs and PEI-MSNPs displaying similar intracellular distributions and co-localization coefficients (Fig. 4a,d,j). In stark contrast, after 24 h, PEI-MSNPs are roughly equally distributed between cytoplasm and acidic cellular compartments (Fig. 4e). The associated mean PCC value drastically decreases from 0.64 (3 h) to 0.36 (24 h), implying a reduced linear correlation between the fluorescence signal of PEI-MSNPs and endo-/lysosomes. At this time point, a high heterogeneity in the intracellular localization was observed between different cells, explaining the large standard deviation (SD) of the mean PCC value. As depicted in Fig. 4f, after 48 h the majority of PEI-MSNPs are excluded from the acidic compartments, with PCC value dropping to 0.25. The ability of PEI-coated particles to escape from the acidic vesicles is attributed to the proton sponge effect of this polymer, which results in the rupture of the membrane organelles^42^. It is important to mention that the possible proton sponge effect of PEI does not change the pH of the endo-/lysosomes^57^, and has no effect on the staining of these organelles with LysoTracker probes. Consequently, a lower co-localization with the LysoTracker Red® can be directly linked to endo-/lysosomal damage and/or rupture.

A similar trend was observed with the multifunctionalized HAPEI-MSNPs. After 48 h the majority of the particles with a HA-PEI bilayer were not co-localized with acidic cellular compartments (Fig. 4i).

However, the initial uptake and endosomal escape rate is very different. At 3 h of incubation, a considerable fraction of HAPEI-MSNPs had already escaped the acidic vesicles (Fig. 4g, mean PCC = 0.45), indicating an effect of the polymeric bilayer in the endosomal escape rate (PCC similar to that of PEI-MSNPs after 24 h, Fig. 4j). The fraction of particles co-localizing the acidic compartments markedly decreased after 24 h, when most HAPEI-MSNPs were found to be no longer entrapped inside the endo-/lysosomal vesicles (Fig. 4h). After 48 h, practically all HAPEI-MSNPs were distributed in the cytosol (Fig. 4i, mean PCC = 0.10), indicating a highly effective escape of HAPEI-MSNPs from the acidic compartments.

The results obtained with fluorescence imaging were further validated by electron microscopy. For the TEM measurements, cells were incubated with differently functionalized particles for 3 h and fixed after 24 h (more info in SI, Supplementary Figs S4 and 5). In agreement with the fluorescence images acquired at this time point, TEM images show that bare MSNPs were clearly trapped inside the lysosomes, MSNPs coated with PEI alone were found to be distributed either in the cytoplasm or inside the endo/lysosomes, and MSNPs with a polymeric bilayer (PEI and HA) were detected mainly outside of the lysosomes (Supplementary Fig. S5).

These results constitute the first irrefutable evidence that coating of nanoparticles with specific polymers induces the rupture of the endo-/lysosomes and further escape to the cytosol. Both fluorescence and electron microscopy images demonstrate an evident enhancement/acceleration of the endosomal escape efficiency with HA-PEI coating compared to using PEI alone. Further research is necessary to assess the mechanism behind this effect, although we speculate that it might be associated either to a faster uptake rate of the HAPEI-coated particles, thanks to the HA targeting, or, more generally, to the presence of an additional polymer. At low pH, the inclusion of an extra polymeric layer can, indeed, increase both the buffering capacity and the polymeric swelling, contributing to the destabilization of the endo-/lysosomal membrane^43^.

### Drug release *in vitro*

Thanks to the therapeutic effectiveness towards a wide range of cancers (carcinomas, sarcomas and hematological cancers)^58^ and to its fluorescent properties^59^, doxorubicin (Dox) was selected as anticancer drug model for the current work. To be efficient, DDSs should guarantee a stable encapsulation of the drug, combined with a controlled release at the specific target. For bare MSNPs, the environmental pH plays a crucial role on the drug release kinetics. Further information about the mechanism of Dox uptake and release in/from MSNPs is reported in SI (Supplementary Fig. S9). Gao *et al*. have shown that the release rate of Dox *in vitro* is accelerated at acidic pH, although a relatively smaller amount can be released at neutral pH as well^60^. In the particle design proposed here, in addition to confer to the MSNPs active targeting towards cancer cells and the capability to induce endosomal rupture, the HA-PEI polymeric bilayer will function as a capping agent, preventing the leakage of the drug before reaching the intracellular environment. At neutral pH, according to the pK_a_ values of PEI and silica hydroxyl groups^53,61^, the electrostatic interactions guarantee a stable attachment of the PEI shell to the particles, impeding the discharge of Dox in blood circulation. At acidic pH, instead, as the majority of the hydroxyl groups of the silica particles are protonated, the electrostatic interactions are minimized, reducing the capping effect of the polymeric coating and facilitating the drug release in the cellular acidic compartments. In this context, Meng and co-workers reported that PEI coating does not hinder the Dox release at acidic pH^47^.

**Figure 5:**
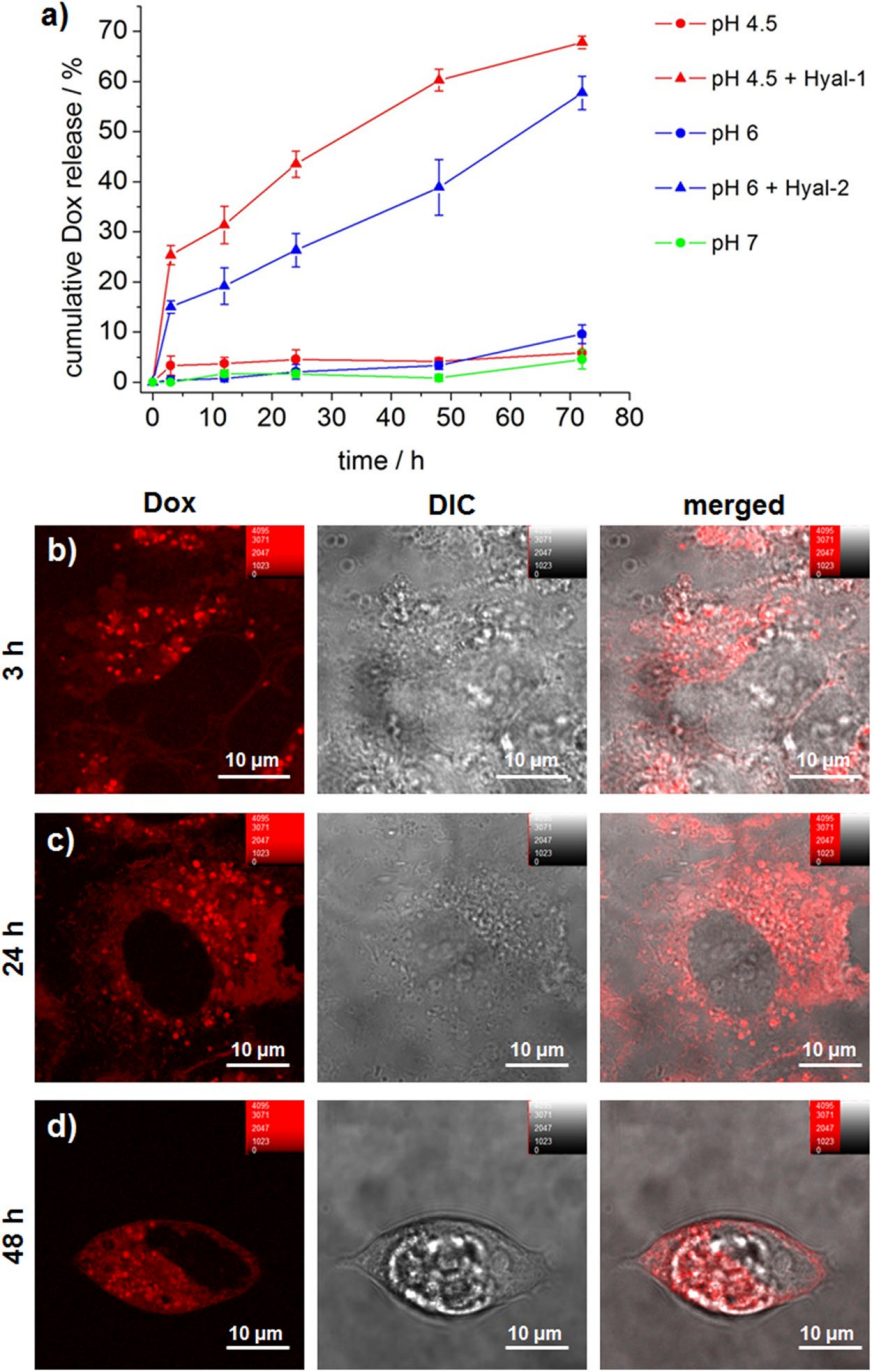
Drug release *in vitro* and *in cellulo*. (**a**) Time-dependent *in vitro* release profile of Dox from HAPEI-MSNPs_Dox (0–72 h) at pH 4.5 (red circle), pH 4.5 + Hyal-1 (red triangle), pH 6 (blue circle), pH 6 + Hyal-2 (blue triangle) and pH 7.4 (green triangle) (each point consists of mean ± SD, n = 3). (**b**–**d**) Fluorescence images of Dox released from HAPEI-MSNPs_Dox inside A549 cells after 3, 24 and 48 h (b-d, respectively). Dox channel (in red), DIC (gray) and merged images are shown from left to right, respectively. The contrast of the red channel was kept constant in all images.

In order to evaluate the capping effect of the polymeric HA-PEI bilayer proposed here, the release kinetics of Dox from HAPEI-MSNPs was estimated *in vitro*. As depicted in Fig. 5a, functionalization of MSNPs with a HA-PEI bilayer resulted in particles with no drug release in both neutral (pH 7) and acidic environments (pH 6 and 4.5). This suggests that the stability of the shell is enhanced by the external HA layer, which likely hinders the polymer detachment, making the coating more compact and stable, even at acidic pH, thanks to the amide bond links to the HA.

In the cellular environment, the external HA shell can be degraded by digestive intracellular enzymes, thereby promoting the discharge of the drug exclusively within the target cell. The main HA digestive enzymes are Hyaluronidase-1 (Hyal-1), which is normally located inside endosomes and lysosomes, and Hyaluronidase-2 (Hyal-2), mainly present on the plasma membrane^62,63^. While most degradation occurs in the acidic compartments, Hyal-2 can already degrade the high molecular weight HA into smaller fragments during the ligand-receptor binding, immediately prior to endocytosis^63^.

Enzyme-mediated HA degradation and subsequent drug release was evaluated by incubating the Dox loaded HAPEI-MSNPs in different solutions at 37 °C. MES buffer (pH 6) with Hyal-2 was selected to mimic the extracellular matrix in tumor tissue, and acetate buffer (pH 4.5) containing Hyal-1 was used to simulate the late endosomes and lysosomes.

The amount of Dox released at different incubation times (3, 12, 24, 48, 72 h) is shown in Fig. 5a. While in absence of enzymes and independently of the pH the percentage of Dox released was negligible, upon enzymatic digestion by Hyal-1 (pH 4.5) or Hyal-2 (pH 6), the release profiles were similar to those of bare MSNP (Supplementary Fig. S6). Similarly to bare MSNPs, Dox release kinetics were faster at more acidic pH, which is in agreement with previous reports^43^. The addition of Hayl-2 to the solution mimicking the extracellular matrix (pH 6) led to a total release of Dox from HAPEI-MSNPs of 58 ± 3% after 72 h. Notably, after only 3 h, 15% of the drug had been already released, suggesting that a partial digestion of HA on the plasma membrane can facilitate some Dox release. The addition of Hyal-1 at 4.5 pH (similar to the endo-/lysosomes) turned out to be the condition with the higher amount of Dox released, reaching a percentage of 68 ± 1% in 72 h. The Dox release profile from HAPEI-MSNPs in the presence of hyaluronidase demonstrates that only enzyme-mediated degradation of the polymeric coating, which occurs exclusively in the cellular environment, triggers drug release from the particles.

### Drug release *in cellulo*

In order to evaluate drug release kinetics *in cellulo*, HAPEI-MSNPs loaded with Dox were added to A549 cells and intracellular Dox release was monitored using fluorescence microscopy (Fig. 5b–d). When adding pure Dox to cells, fluorescence could be detected uniformly in the cytoplasmic region after 24 h, with no signal coming from the cell nucleus (Supplementary Fig. S7). The absence of fluorescence in the cell nucleus is associated to Dox intercalation between the DNA base pairs. As reported by several research groups, nuclear penetration causes a drastic quenching of Dox fluorescence^64–66^, up to 95% of its intrinsic emission^67^.

Similar to the pure drug, after 3 h of incubation with Dox-loaded HAPEI-MSNPs, fluorescence could be detected in the cytoplasm of A549 cells. The weak dispersed signal in the cytoplasmic area was attributed to a small ratio of Dox release within the 3 h of incubation (which is in agreement with the results obtained *in vitro* in the presence of HA-degrading enzymes). While cells incubated with the pure drug only show a disperse fluorescence over the whole cytoplasmic region (Supplementary Fig. S7), when Dox-loaded nanoparticles are used, it was possible to observe bright dots in the intracellular environment (Fig. 5b). These bright dots were attributed to the HAPEI-MSNPs containing Dox. At longer time intervals (24 and 48 h), the fluorescence signal from Dox was more intense over the cytoplasm, while the bright spot-like signals arising from the particles became dimmer (Fig. 5c,d). This suggests that during time Dox was released from the particles into the intracellular environment (note that after 3 h the cells were washed, stopping further uptake of any drug and/or particles). This change in the distribution of Dox fluorescence signal was observed in all cells (Supplementary Fig. S8) and is in agreement with the enzyme-mediated release profile obtain in the *in vitro* experiments.

### Anticancer efficiency: cell viability tests

**Figure 6:**
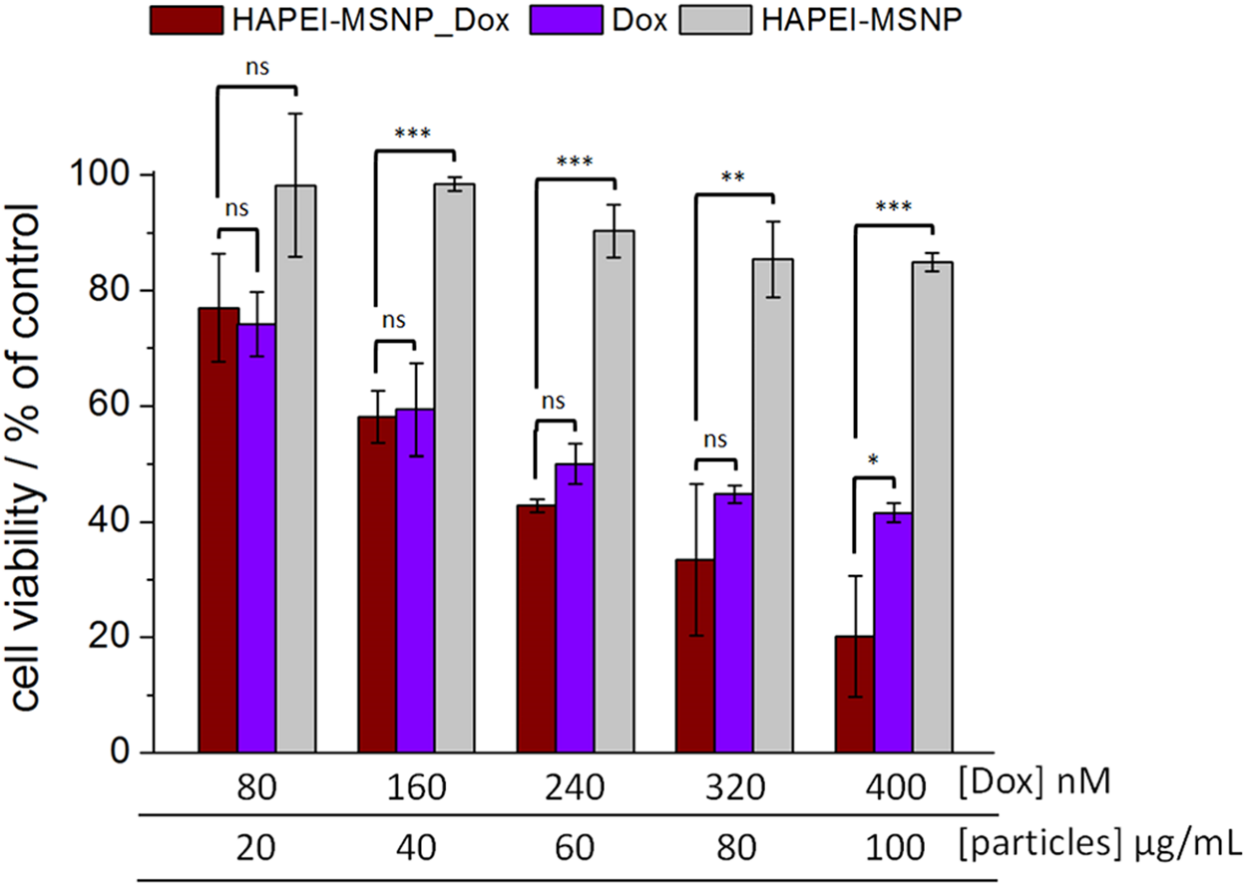
Anticancer efficiency of Dox-loaded HAPEI-MSNPs. Viability tests of A549 cells incubated with HAPEI-MSNPs_Dox (wine red line column), free Dox (violet column) and empty HAPEI-MSNPs (light gray) for 72 h. Aliquots of 2, 4, 6, 8, 10 μL corresponding to final Dox concentrations of 80, 160, 240, 320, 400 nM and particle concentration of 20, 40, 60, 80, 100 μg/mL, respectively, were added to 1 mL of cell culture medium. All data are shown as mean ± SD (n = 3) with ns = (p > 0.05), *(p < 0.05), **(p < 0.01) and ***p < 0.001).

In order to evaluate the efficiency of the newly developed polymer-coated particles as anticancer DDSs, we monitored the cell viability 72 h after treatment with free Dox, Dox-loaded HAPEI-MSNPs and empty HAPEI-MSNPs, at different concentration of drug/particles (Fig. 6).

While at low concentration (<40 μg/mL) empty HAPEI-MSNPs are not toxic, higher concentration leads to a lower cell viability, reaching a minimum of 85% at 80–100 μg/mL. A similar effect of empty HA-coated MSNPs on the cell viability was reported by previous studies^23^. In our experiments, it can be associated either to the massive uptake of HA-coated particles into A549 cells (as shown in Fig. 3c, above) or to the intrinsic toxicity of PEI itself ^68^. Note that the HAPEI-MSNP uptake is highly specific towards CD44-overexpressing cancer cells and, consequently, the limited toxicity of the empty particles cannot be considered of negative impact on the functionality and effectiveness of the DDS.

The cytotoxicity of Dox-loaded HAPEI-MSNPs is at least as high as the one of free Dox, indicating an efficient intracellular release and trafficking of the drug. For free and Dox-encapsulated particles, the cell viability decreases with the increase of the drug concentration, reaching a mean value of 42 and 20% at 400 nM, respectively. As a general trend, Dox encapsulated in HAPEI-MSNPs seems to induce higher cell mortality rate in comparison to free Dox, with a sharper discrepancy at high drug/particle concentration. This enhanced killing capability might be explained by the well-engineered properties of the DDS (high uptake, endosomal escape and controlled drug released) allied with the toxicity co-effect of the nanocarrier itself.

Taken together these results demonstrate that our DDS has great therapeutic potential, specifically towards CD44-overexpressing cancer cells, having a comparable, or better, efficiency than free Dox. The high therapy efficiency achieved at low drug concentrations is strictly related to the fast internalization rate (HA coating targeting effect), to the improved endosomal release, with consequent retention of the particles in the cytoplasm (HA-PEI shell inducing endosomal rupture), and to a controlled drug release overtime (enzymatic polymeric digestion).

In conclusion, in this work, a polymeric bilayer functionalization of mesoporous silica nanoparticles was designed for drug delivery-based tumor therapy. Hyaluronic acid and polyethylenimine layers provide the drug delivery system with active targeting and endosomal escape capability, simultaneously, enhancing the therapeutic efficiency. The as-obtained nanoparticles (HAPEI-MSNPs) turned out to possess an excellent active targeting capability towards CD44-overexpressing cell (A549). Furthermore, the presence of PEI was demonstrated to trigger endosomal escape of both PEI-MSNPs and HAPEI-MSNPs. Unlike previous reports, the endosomal breakout of PEI- and HAPEI-coated mesoporous silica nanoparticles was unambiguously shown. Fluorescence data were consistent with the results obtained by electron microscopy. A time-lapse fluorescence-based investigation showed that after 48 h, multifunctionalized particles were mainly localized in the cytoplasm (data confirmed by PCC analysis). Additionally, the system enabled Dox release upon HA degradation by specific enzymes, proving the capping effect of the polymeric shell and the enzyme-responsive intracellular drug release. Dox-loaded HAPEI-MSNPs exhibit great killing efficiency at low drug concentrations (nM range), which was comparable with that of pure Dox, but with specificity towards CD44-overexpressing cancer cells. These results provide evidence that the DDS here developed, supplied with targeting capability towards cancer cells, endosomal escape capacity, controlled drug release and, consequently, high therapeutic effect, holds great potential for tumor therapy applications. The polymeric functionalization proposed can be applied to a wide range of nanocarriers towards the increase of the therapeutic power at low drug dose and the decrease of exocytosis rate, drastically reducing the side effects of anti-cancer drugs.

## Methods

### Materials

Tetraethyl orthosilicate (TEOS, 98%), cetyltrimethylammonium chloride solution (CTAC, 25% in H_2_O), triethanolamine (TEA, 99%), hydrochloric acid (HCl, 1 N), rhodamine B basic violet 10 (RhodB, 93%), fluorescein 5(6)-isothiocyanate (FITC, ≥90% HPLC), polyethyleneimine solution (PEI, 50% w/v in H_2_O), N-(3-dimethylaminopropyl)-N′-ethyl-carbodiimide (EDC, 97%), N-hydroxysulfosuccinimide sodium salt (sulfo-NHS, ≥98% HPLC), doxorubicin hydrochloride (Dox, suitable for fluorescence, 98–102%, HPLC), sodium acetate buffer solution, MES hydrate (titration, ≥99.5%), hyaluronidase type I-S (Hyal-1, from bovine testes), hyaluronidase Type II (Hyal-2, from sheep testes) were purchased from Sigma Aldrich. Sodium hyaluronate (HA, research grade, 289 kDa) was obtained from LifeCore BioMedical. Dulbecco’s modified eagle medium (DMEM), and Lysotracker RED DND-99 were purchased from Molecular Probes. Gentamicin, Dulbecco’s phosphate buffered saline (PBS, no calcium, no magnesium), Hank’s balanced salt solution (HBSS, no phenol red), GlutaMaxi supplement, fetal bovine serum (FBS, South America origin), Ethanol (absolute, 99.9%), Vybrant DiO cell-labeling solution were purchased from ThermoFisher Scientific. Trypan blue solution (0.4%, TC grade) was purchased from Life Science. All the chemicals were used without further purifications.

### HAPEI-MSNPs preparation

The basic synthesis of MSNPs was performed by mixing 0.18 g of TEA with a solution containing 24 mL of CTAC and 36 mL of milli-Q. The mixture was then heated to 60 °C. After 1 h, 20 mL of TEOS (20 v/v % in 1-octadecene) was gently added. The reaction was kept on going overnight at 60 °C under magnetic stirring. After cooling to room temperature, the particles were washed with a solution of HCl 1.1 M in water/ethanol (v/v = 1.25:10) using centrifugation-dispersion-sonication cycles in order to remove CTAC from the pores. Subsequently, two washing cycles were performed with milli-Q to bring the solution to neutral pH. At this step, the concentration of particles in the colloidal solution was estimated to be 10 mg/mL. The loading of RhodB and FITC were performed in milli-Q, while for the Dox soaking, MSNPs were first dispersed in phosphate buffer (pH 9) to increase the loading efficiency, which was otherwise minimized at neutral pH. The three mixtures were left under magnetic stirring (500 rpm) overnight. After centrifugation, the supernatants were then replaced with milli-Q, obtaining MSNPs_RhodB/FITC/Dox as pellet (MSNPs_X). A solution 0.75% PEI in H_2_O was adjusted at pH 7 and consequently added dropwise to loaded-MSNPs in milli-Q water (1:1 v/v). PEI layer formation takes about 3 h under magnetic stirring. In the meanwhile, HA were dissolved in 10 mL of MES buffer (0.1 M, pH 6) (to be 0.4 mM of the final HA concentration) and stirred for few hours. Afterwards, EDC and NHS-sulfo were simultaneously added to the HA solution (with a final concentration of 1 mg/mL of EDC and NHS-sulfo) and kept under stirring for 30 minutes for the activation of the carboxylic groups. On the other hand, PEI functionalized particles (PEI-MSNPs_X) were washed via centrifugation and dissolved in MES buffer as well. As a final step, 1 mL of carboxyl-activated HA was slowly added to 3 mL of PEI-MSNPs_X (1 drop/5 s) and kept under stirring overnight. The products (HAPEI-MSNPs_X) were then washed and dispersed in milli-Q. Supernatants of each washing step were collected and analyzed with UV-VIS spectrometer (Lambda 950, PerkinElmer) in order to estimate the soaked cargo concentrations via absorbance. Final concentrations of soaked RhodB and FITC inside HAPEI-MSNPs were determined to be 276 and 165 μM, respectively. Dox concentration in HAPEI-MSNPs used for the viability experiments was 40 μM, while for the release experiments (*in vitro* and *in cellulo*) a higher concentration was used for facilitating the absorbance and emission detection (150 μM).

### HAPEI-MSNPs_X characterization

MSNPs obtained by biphase stratification method were first characterized for size, shape and porosity by transmission electron microscopy (TEM). The colloidal solution was deposited on an amorphous carbon-coated copper grid and measured by JEOL-JEM 2100 TEM (200 kV). The loading was verified by collecting wide-field images and the relative emission spectrum of MSNPs_Dox/RhodB/FITC. These measurements were recorded by using an inverted optical microscope (TiU, Nikon). Argon krypton ion laser (488 nm) was used for FITC and Dox excitation, while Nd:YAG laser (532 nm) was applied on RhodB samples. Lasers were focused on the samples by a 100x oil-objective (N.A. 1.3, plan fluo Nikon) for RhodB and Dox samples, while a 40x objective (N.A. 0.6, plan fluo Nikon) was used for the FITC sample. Emission images were recorded by a charge-coupled device (CCD) camera (ImagEM, Hamamatsu) operating at −85 °C. The spectra were collected by a CCD camera (DU920P, Andor), equipped with a spectrograph (iHR320, Horiba), operating at −85 °C. A pinhole (100 μm) was placed before the entrance of the spectrograph. Longpass filters (HQ500LP for 488 nm, HQ545LP for 532 nm excitation, Chroma) were employed either in front of the imaging CCD or in front of the spectrograph to block the excitation light. The zeta potential of nanoparticles was measured in milli-Q water by Delsa Nano HC (Beckman Coulter) and FE-SEM images were collected by Quanta FEG250 FEI.

### Cell culture

All cells were cultured in 25 cm^2^ cell culture flasks at 37 °C and under humidified 5% CO_2_ atmosphere. The cell passage was performed via trypsinization every 2–3 days, when the confluency reached 80%. NIH3T3 and A549 cell lines were maintained in DMEM medium containing 10% FBS, 1% L-glutammax and 0.1% gentamicin. For confocal imaging, cells were cultured in 35-mm glass bottom dishes (MatTeK). When the confluency reached about 60%, the medium was replaced with fresh medium (1 mL) and particles were added into the dishes, which were then incubated at 37 °C under humidified 5% CO_2_ for different time intervals.

### Cellular uptake

For verifying the effect of different functionalization on the uptake, RhodB-loaded particles (MSNPs_RhodB, PEI-MSNPs_RhodB and HAPEI-MSNPs_RhodB) were incubated with NIH3T3 and A549 cells for 3 h at 37 °C. Before the fluorescence measurements, the dishes were washed three times with PBS to remove the residual extracellular particles; the cells were then kept in HBSS. The plasma membrane was stained with DiO (1 μM) in HBSS for 15 min and the dishes were visualized under a confocal fluorescence microscope (FV1000, Olympus). High magnified images were obtained with 100x oil objective (N.A. 1.40). The RhodB-encaspulated particles were visualized with 561 nm (20 μW, power density ~15.8 kW/cm^2^) excitation wavelength, while DiO was imaged by using a 488 nm laser (5 μW, power density ~5.3 kW/cm^2^). A DM 405/488/559/635 was chosen as the main dichroic mirror; emissions were detected through bandpass filters (BA 500–520 and BA570–670, respectively). A SDM-560 was placed in front of the DiO detection channel as sharp-cut dichroic mirror for splitting the emissions. The Z-stack method, changing the focal length from bottom to top of a single cell, was used to collect a set of images. The Z-reconstruction then offers an orthogonal view of the cell thickness cross-section on the x/y-axis as a clear proof of the particles internalization. Images were processed using FV10-ASW Viewer Software. Fluorescence intensity analysis was performed by MATLAB software in order to estimate the mean intensity per μm^3^ of cell. After background subtraction, a single-cell area was manually selected and the cell volume was calculated over a constant height of 1 μm (starting from the bottom of the cell).

### Intracellular trafficking: fluorescence microscopy

In order to track the intracellular fate of the particles over time, FITC was employed as cargo. MSNPs_FITC, PEI-MSNPs_FITC, HAPEI-MSNPs_FITC were incubated individually with A549 cells for 3 h. Before the measurements, the dishes were washed with PBS three times to remove the residual extracellular particles; the cells were kept in HBSS during the measurements. Confocal fluorescence measurements were performed immediately after the washing, obtaining data of 3 h of incubation. On the other hand, after washing with PBS, other copies of the same samples were suspended in fresh medium and placed back in the incubator for measurements after 24 and 48 h. For the acidic compartment imaging, endo/lysosomes were stained with Lysotracker Red (50 nM) in HBSS for 15 mins. Confocal images were obtained with FV 1000 Olympus microscope, using 100x oil objective (N.A. 1.40). 488 nm (5 μW, ~5.3 kW/cm^2^) and 561 nm (2 μW, power density ~1.6 kW/cm^2^) excitation wavelengths were applied for detecting FITC-loaded particles and Lysotracker-stained endo-/lysosomes emissions, respectively. A DM 405/488/559/635 was chosen as main dichroic mirror; emissions were detected through bandpass filters (BA 510–530 and BA575–675, respectively). Short-cut dichroic mirrors were located in the emission pathways to split the emissions. A SDM 510 was placed before the detection channels to reject scattered and excitation light. A SDM 530 was applied to selectively reflect the FITC emission towards the 488-channel. Intracellular location of the particles was verified collecting high magnification 2D-confocal fluorescence images. Images were processed using FV10-ASW Viewer Software. Pearson correlation coefficient (PCC)^56^ analysis was carried out using MATLAB software. After background subtraction and single cell area selection, PCC values were estimated by the following equation (Eq. 1)

1 $$PCC=\frac{{\sum }_{i}({R}_{i}-\bar{R})\cdot ({G}_{i}-\bar{G})\,}{\sqrt{{\sum }_{i}{({R}_{i}-\bar{R})}^{2}\cdot {\sum }_{i}{({G}_{i}-\bar{G})}^{2}\,}},$$

where *R*_*i*_ and *G*_*i*_ are the intensities per pixel *i* of the red and green channel, respectively, and $\bar{R}$ and $\bar{G}$ are the corresponding mean intensities over the cell area.

PCC values near 0 indicate that the fluorescence intensities of the two channels are uncorrelated, while PCC values are close to 1 when the two fluorescence intensities are perfectly linearly related. Threshold PCC values of the current study, related images and descriptions are reported in SI (Supplementary Fig. S3).

### Drug release *in vitro*

In order to perform the *in vitro* follow-up of the Dox release, three different pH buffers were selected: acetate buffer (pH 4.5), MES buffer (pH 6) and PBS (pH 7.4). Dox-incorporated particles were suspended in the different buffers individually and kept in a thermomixer (thermomixer comfort, eppendorf) under stirring (300 rpm) at 37 °C. In order to check the enzymatic digestion of HA layer, Hyal-1 and Hyal-2 (150 U/mL) were added to HAPEI-MSNPs_Dox in acetate and MES buffer, respectively. Aliquots of the suspensions were taken at different incubation time (0–72 h), centrifuged and analyzed in the micro-volume spectrophotometer (BioDrop μLITE, BioDrop). The concentration of the Dox released was estimated by collecting the absorbance at 490 nm.

### Drug release *in cellulo*

10 μL of HAPEI-MSNPs_Dox (Dox: 150 μM) were added to 1 mL of medium containing A549 cells on a glass-bottom dish. The dish was then placed in the incubator for 3 h. Subsequently, the un-internalized particles were washed away with PBS washing (x3) and the cells were measured by using a confocal microscope (FV 1000 Olympus microscope) without any staining, obtaining data of 3 h of incubation. On the other hand, after the PBS washing, other copies of the same samples were suspended in fresh medium and placed back in the incubator for measurements after 24 and 48 h. Differential interference contrast (DIC) images were collected to visualize the cells and Dox emission was detected by using a 488 nm laser (5 μW, power density ~5.3 kW/cm^2^), a 100x oil objective (N.A. 1.4) and a bandpass filter 600–670 nm.

### Anticancer efficiency

In order to carry out viability tests, the cells were seeded in TC Dish 35 Standard dish (SARSTEDT) with 1 mL of medium and growth until density reached about 5 × 10^5^ cells/cm^2^. After removing the dead cells and placing fresh medium, aliquots of 2, 4, 6, 8 and 10 μL solutions containing HAPEI-MSNPs_Dox, free Dox and empty HAPEI-MSNPs were added individually to the culture medium. The corresponding concentrations of Dox and particles in the medium were 80, 160, 240, 320 and 400 nM and 20, 40, 60, 80, 100 μg/mL, respectively. The particle solutions were incubated with the cells overnight, then extracellular particles were removed by PBS washing and a fresh medium was replaced. A further incubation was executed, reaching 72 h in total. The viability was estimated comparing the number of viable cells in the dishes after the treatment (HAPEI-MSNPs_Dox, free Dox and empty particles incubation) with number of viable cells in a control dish (no treatment). The cell counting was performed by trypsinazing the cells and depositing aliquots of the cell suspension in glasstic slide 10 with grids (KOVA). The estimation of viable cell number was accomplished by following KOVA system protocol; non-viable cells were stained with Trypan blue solution to be excluded from the counting. The viability data were expressed as mean percentage of viable cell compared to control.

### Statistical analysis

Data were shown as mean ± standard deviation. Each experiment was repeated at least 3 times (n ≥ 3, specific n values indicated in figure captions). One-way ANOVA test was performed to compare differences among groups, followed by *post-hoc* t-test analysis. Results were considered statistically significant at p < 0.05.

## Supplementary information

Supplementary Information

## Data Availability

The datasets generated during the current study are available from the corresponding authors on reasonable request.
