# Supplementary material for: Polymeric Engineering of Nanoparticles for Highly Efficient Multifunctional Drug Delivery Systems
Source: Sci Rep. 2019 Feb 25;9:2666. doi: 10.1038/s41598-019-39107-3 (PMC6389875; doi:10.1038/s41598-019-39107-3)
Supplement: Supplementary file 1 — Supplementary Information [file 41598_2019_39107_MOESM1_ESM.pdf]

Supporting Information

Polymeric Engineering of Nanoparticles for Highly Efficient Multifunctional Drug Delivery Systems

Beatrice Fortuni*[1], Tomoko Inose[2], Monica Ricci[1], Yasuhiko Fujita[3], Indra Van Zundert[1], Akito Masuhara[4], Eduard Fron[1], Hideaki Mizuno[1], Loredana Latterini[5], Susana Rocha*[1] and Hiroshi Uji-i*[1,2]

[1]KU Leuven Celestijnenlaan 200G-F 3001 Heverlee, Belgium
[2]RIES Hokkaido University, N20W10 Kita-Ward Sapporo, 0010020, Japan
[3]Toray Research Center, Inc., 3-3-7, Sonoyama, Otsu, Shiga 520-8567, Japan
[4]Yamagata University, Yonezawa, Yamagata 992-8510, Japan
[5]University of Perugia, via Elce di sotto 8, Perugia, Italy

***beatrice.fortuni@kuleuven.be**, **susana.rocha@kuleuven.be**, **hiroshi.ujii@kuleuven.be**

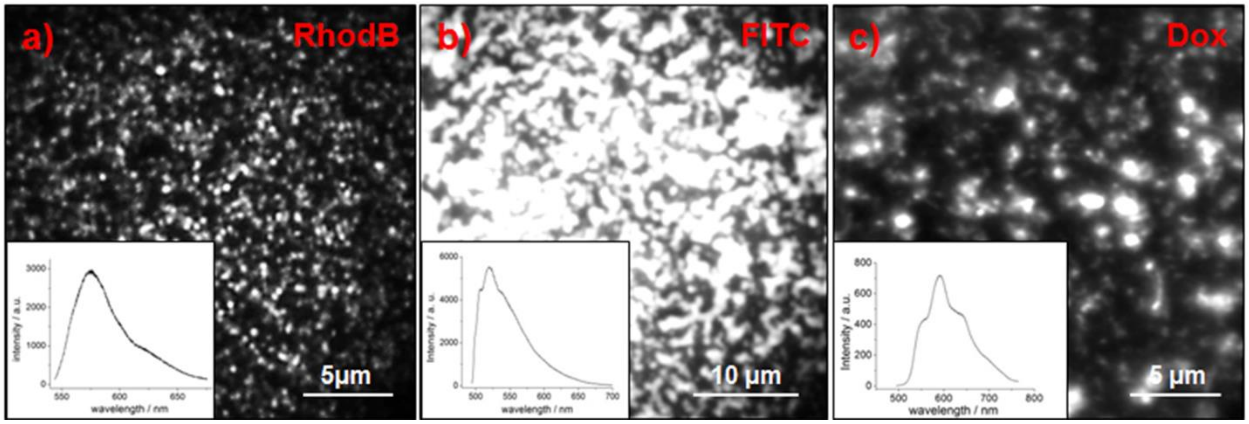

Figure S1. Cargo loading into MSNPs: wide-field fluorescence images (**a-c**) and relative emission spectra (insets) of MSNPs loaded with RhodB (a), FITC (b) and Dox (c). Emission spectra were collected by focusing the laser on one bright spot.

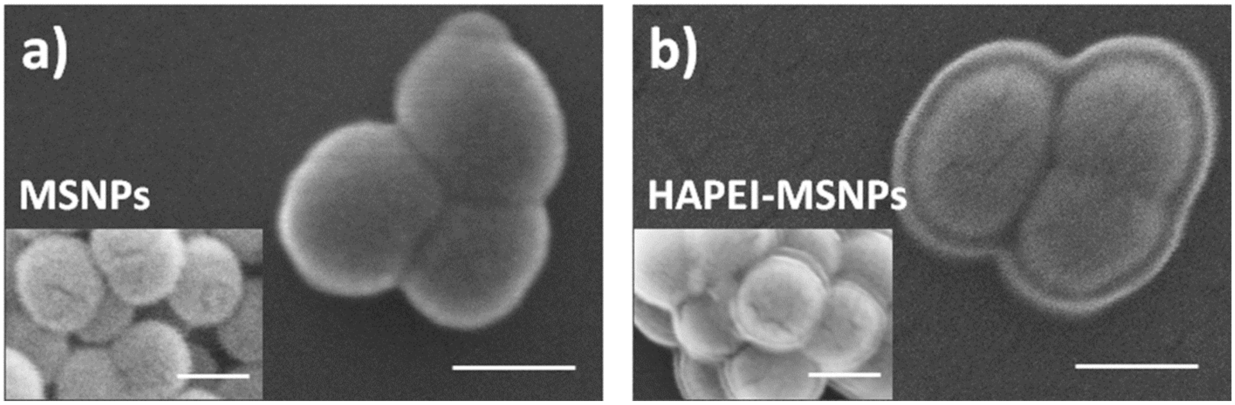

Figure S2. Supplementary FE-SEM images of MSNPs (**a**) and HAPEI-MSNPs (**b**), scale bar: 100 nm.

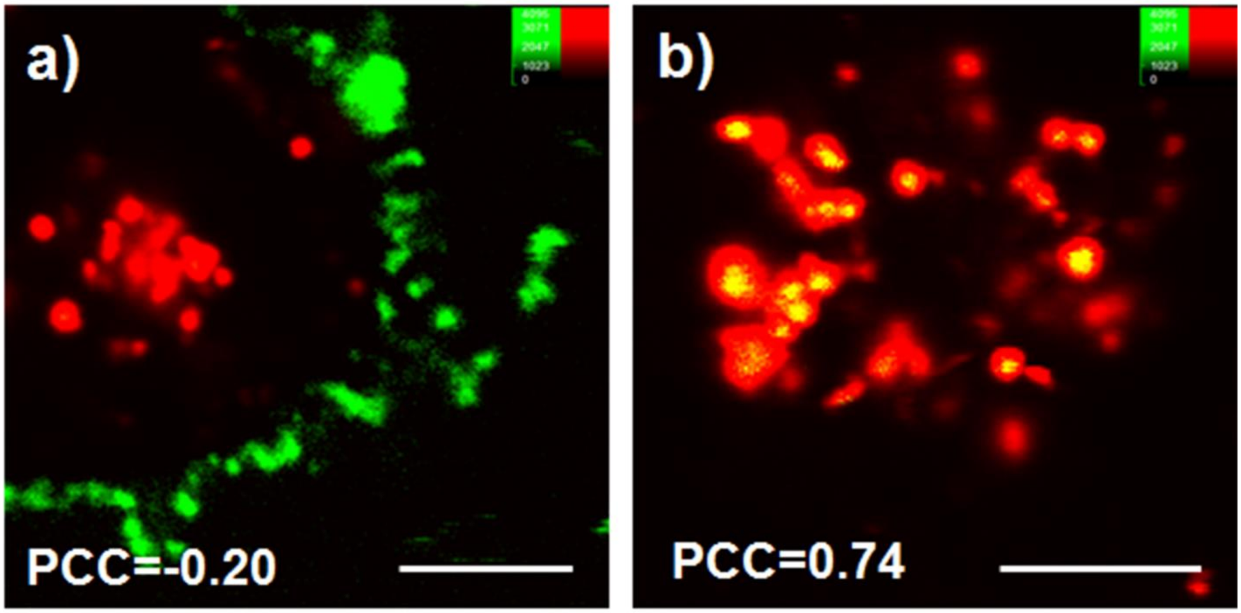

Figure S3. PCC threshold values: fluorescence images representing uncorrelated (**a**) and correlated (**b**) intensity distribution of the two channels (green and red). Scale bar: 5 µm.

PCC threshold values: According to the theory, PCC values can vary from -1, which indicates that the two fluorescence intensities are totally but inversely correlated, to +1 which is associated to a total linear correlation[S1]. Values close to zero are related to an uncorrelation with one another, with no linear or inverse proportionality. In order to establish the threshold values of our measurements, we collected both an image where the two intensities were totally excluded, and an image with the maximum co-localization detected. Figure S3a was recorded instantaneously after the addition of PEI-MSNP_FITC nanoparticles into the medium containing

A549 cells, under the confocal microscope. This image is taken as a reference for minimum correlation obtainable in the current experiment, corresponding to a PCC value of -0.20. On the other way, Figure S3b was collected from A549 cells incubated with MSNP_FITC for 3 h. The PCC value of this image was estimated to be 0.74, representing the maximum correlation value reachable in our measurements.

Intracellular trafficking: electron microscopy.

For the TEM measurements, mesoporous silica-coated gold nanoparticles (Au@MSNPs) were used instead of MSNPs in order to ensure the observation of their highly electron dense metal cores. Au@MSNPs were synthesized following the protocol reported by Chen *et al.*[S2]. Briefly, 0.6 mL of 0.5 M NaOH was added to 24 mL of milli-Q water and the solution was used to dissolve 0.05 g of CTAB. After stirring at 80°C for 15 min, 1 mL of formaldehyde aqueous solution (3.7% wt) was added to the mixture, followed by 0.8 mL of 0.05 M $HAuCl_4$ aqueous solution. After 10 min, a solution containing 0.25 g of TEOS and 0.50 g of ethanol was added. After 1 h of reaction, the product was washed with a solution of HCl 1.1 M in water/ethanol (v/v = 1.25:10) using centrifugation-dispersion-sonication cycles in order to remove the surfactant from the pores. Subsequently, two washing cycles were performed with milli-Q to bring the solution to neutral pH. The as-obtained core-shell particles were characterized by TEM (Fig. S4).

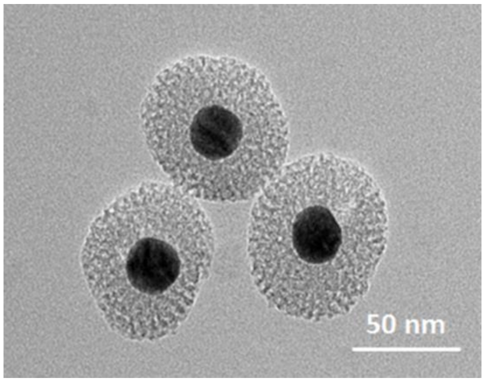

Figure S4. TEM image of Au@MSNPs, which were used for the TEM measurements performed in A549 cells (Fig. S5).

The exact same surface modifications as those used for MSNPs were performed on Au@MSNPs obtaining PEI-Au@MSNPs and HAPEI-Au@MSNPs. In addition, the fluorescence-based tracking experiments performed for HAPEI-MSNPs were also carried out with HAPEI-Au@MSNPs, ensuring their identical behavior (data not shown).

Au@MSNPs, PEI-Au@MSNPs and HAPEI-Au@MSNPs were individually incubated with A549 cells for 3 h. Subsequently, the medium was refreshed to remove excess nanoparticles which were not endocytosed by the cells. After 24 h, cells were fixed using 2.5% glutaraldehyde in phosphate buffer and detached from the bottom of the petri-dish using a cell scraper. The collected cells are kept in the fixative for 24 hours at 4°C. Then, they were washed with 0.1 M cacodylate buffer and post-fixed with 1% osmium tetroxide in the same buffer containing 1.5% potassium ferricyanide for 1 h and 30 min at 4°C. Next, the samples were embedded in agarose

and dehydrated in ethanol, infiltrated with an Epon resin for two days, embedded in the same resin and polymerised at 60°C during 48 h. Ultrathin sections were obtained using the Ultracut UCT ultramicrotome (Leica microsystems) and mounted on Formvar-coated copper grids. They were stained with 2% uranyl acetate in water and lead citrate. Images of the sections (Fig. S5) were acquired by a Tecnai T12 electron microscope equipped with an Eagle 4kx4k CCD camera (Thermo Fisher Scientific, The Netherlands).

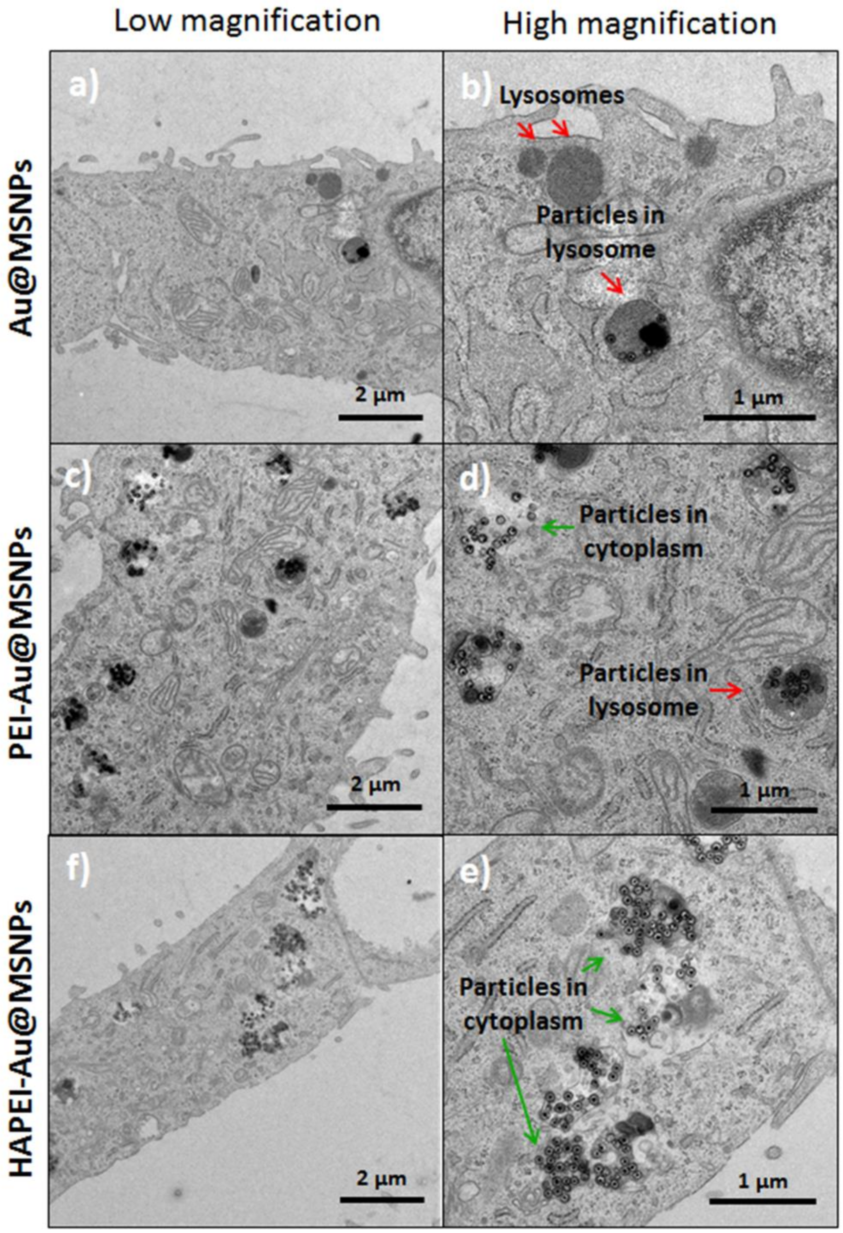

Figure S5. Influence of surface modification on the intracellular trafficking: TEM images of A549 cells after 24 h of incubation with Au@MSNPs (**a,b**), PEI-Au@MSNPs (**c,d**) and HAPEI-Au@MSNPs (**e,f**), respectively.

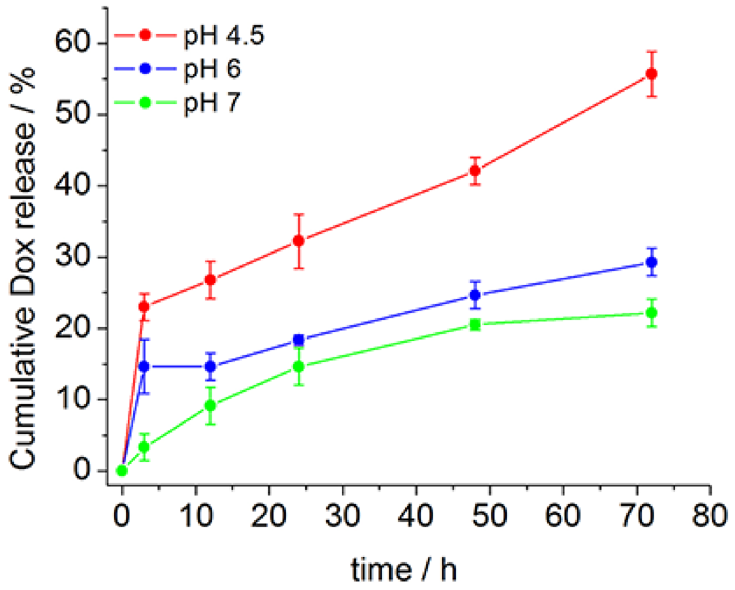

Figure S6. Time-dependent release profile of Dox from MSNP_Dox (0-72 h) at pH 4.5 (red circle), pH 6 (blue circle) and pH 7.4 (green circle) (each point consists of mean ± SD, n=3).

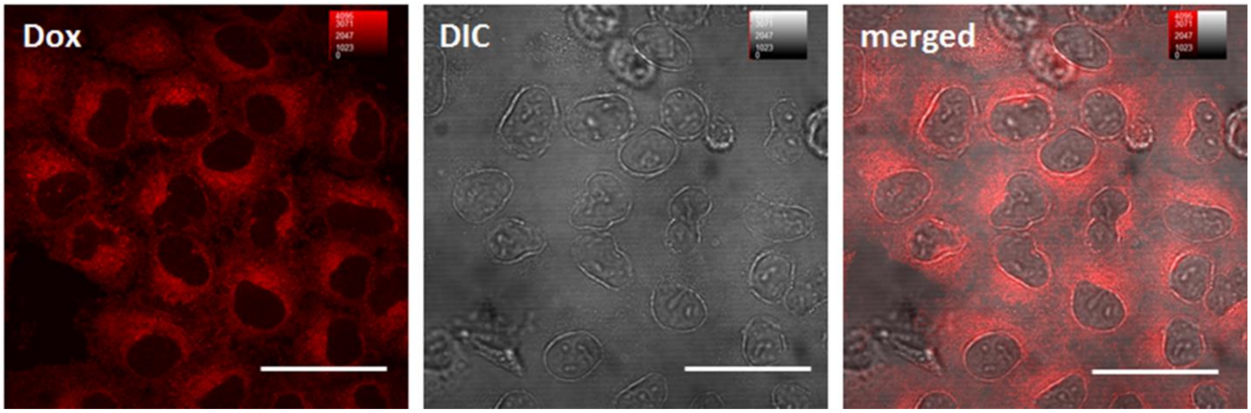

Figure S7. Intracellular fluorescence distribution of pure Dox: low magnification fluorescence images of Dox inside A549 cells after 24 h of incubation. Dox channel, DIC and merged images are reported from left to right, respectively. Scale bar: 40 µm.

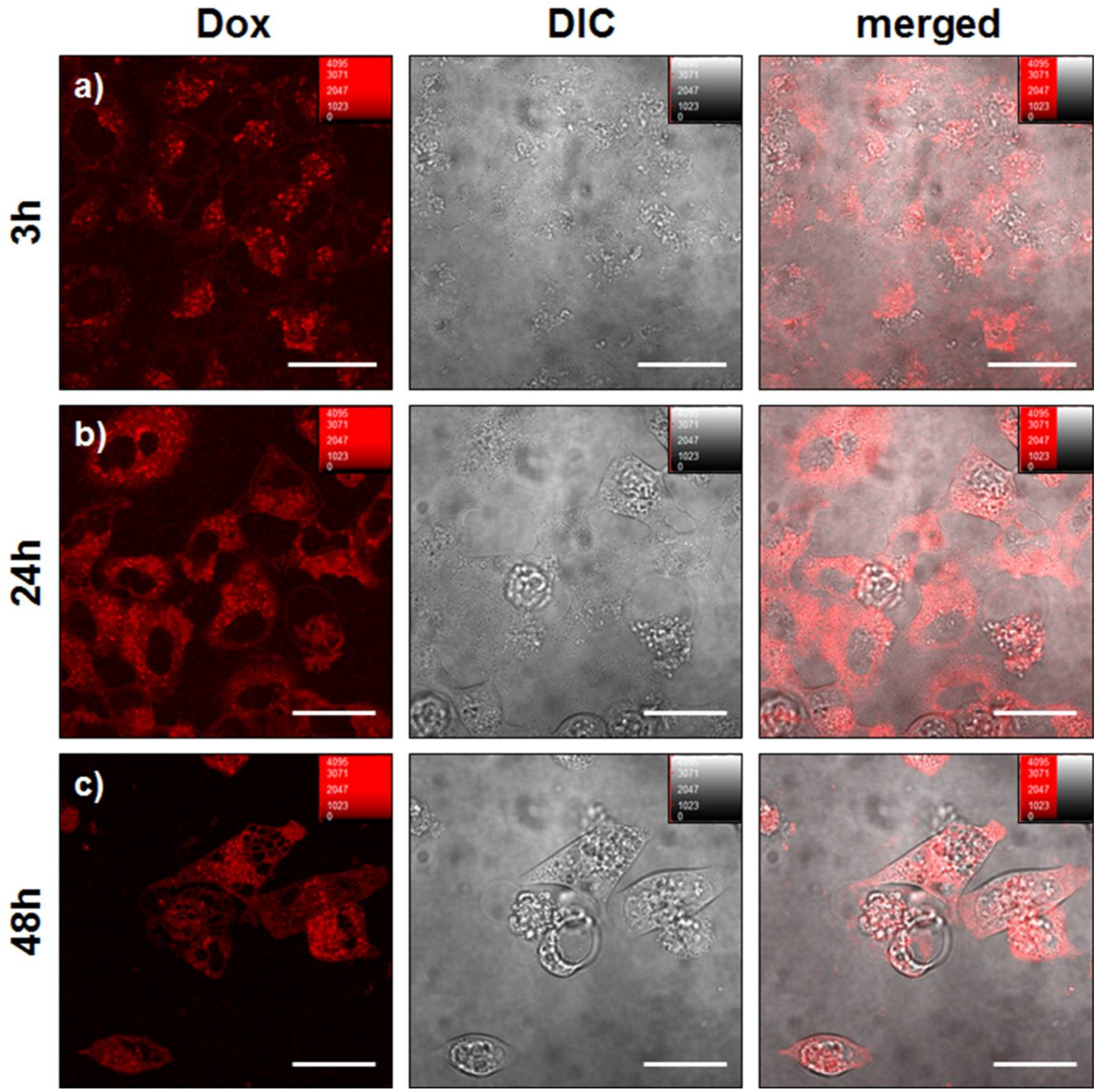

Figure S8. Drug release *in cellulo*: low magnification fluorescence images of Dox release from HAPEI-MSNP_Dox inside A549 cells after 3, 24 and 48 h incubation (**a-c**, respectively). Dox channel, DIC and merged images are reported from left to right, respectively. Scale bar: 30 µm.

Doxorubicin uptake and release mechanism

The uptake and release rate of a molecule inside/from MSNPs depends both on the electrostatic interactions with the silica surface and on its solubility. Consequently, the diffusion of molecules into the pores is strongly affected by the medium (polarity and pH). In the particular case of Dox, the limited aqueous solubility at neutral pH[S3,4] can be considered the main driving force of the encapsulation. The encapsulation efficiency can be, therefore, maximized by tuning the pH. To this end, the amino group plays a fundamental role; with a pK_a

of 8.2[S5], its deprotonation at basic pH (> 8.2) decreases the solubility of Dox in aqueous media (Supplementary Fig. S9), promoting the accumulation of the molecules inside the particle pores. On the other hand, pK_a values of the remaining –OH groups were estimated to be ≥ 9.5[S5,6], thus, their ionization, occurring exclusively at strongly basic pH, does not affect the molecule solubility at pH ≤ 9.

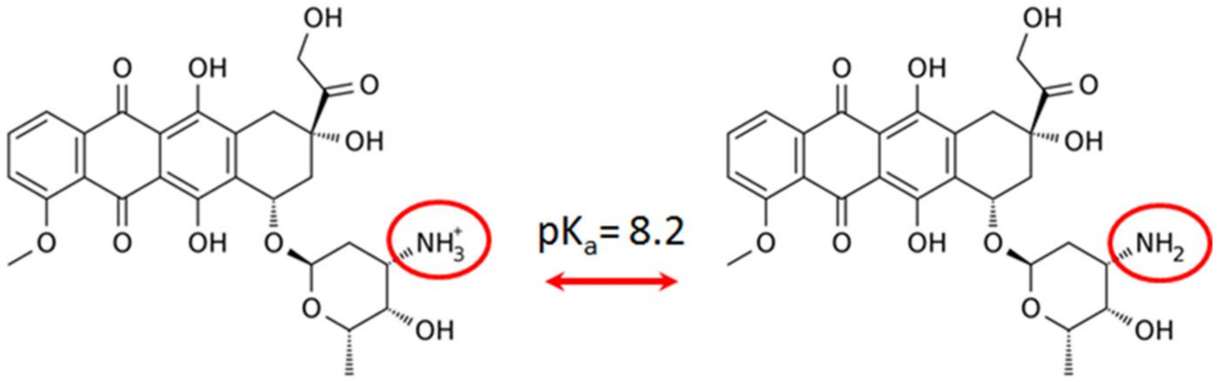

Figure S9. Ionization of the amino group of Dox at pH < 8.2.

The drug release mechanism is mainly associated to the protonation of the silica surface at acidic pH. The percentage of protonated hydroxyl groups increases by decreasing pH, reducing the electrostatic interactions with Dox molecules at acidic pH and, therefore, promoting the drug release (as we demonstrated by *in vitro* experiments, Supplementary Fig. S6).

Based on this information, in order to maximize the encapsulation efficiency, we performed the loading of Dox in MSNPs at pH 9 (phosphate buffer). For maintaining the molecule encapsulated after the loading process, the coating of PEI was performed at pH 7. At neutral pH, despite the increase in Dox solubility, the electrostatic interactions of the amino groups with the silica surface (≡Si-OH, pK_a = 6.8) prevent the drug leakage. The amide bond formation between PEI and HA was carried out in MES buffer (pH 6). Despite the slightly acidic pH used for this step, the drug loss was negligible, probably due to the fast polymeric coating formation and to the short reaction time (3 h). Note that the absorbance of the supernatants after each washing step (centrifugation-dispersion-sonication) was recorded to establish the drug loss and finally estimate the Dox concentration remaining inside the DDS at the end of the fabrication process.

References:

S1 Dunn, K. W., Kamocka, M. M. & McDonald, J. H. A practical guide to evaluating colocalization in biological microscopy. *American Journal of Physiology-Cell Physiology* **300**, C723-C742 (2011).

S2 Chen, J., Zhang, R., Han, L., Tu, B. & Zhao, D. One-pot synthesis of thermally stable gold@ mesoporous silica core-shell nanospheres with catalytic activity. *Nano research* **6**, 871-879 (2013).

S3 Fülöp, Z., Gref, R. & Loftsson, T. A permeation method for detection of self-aggregation of doxorubicin in aqueous environment. *Int. J. Pharm.* **454**, 559-561 (2013).

S4 Fritze, A., Hens, F., Kimpfler, A., Schubert, R. & Peschka-Süss, R. Remote loading of doxorubicin into liposomes driven by a transmembrane phosphate gradient. *Biochimica et Biophysica Acta (BBA)-Biomembranes* **1758**, 1633-1640 (2006).

S5 Skovsgaard, T. Transport and binding of daunorubicin, adriamycin, and rubidazone in Ehrlich ascites tumour cells. *Biochem. Pharmacol.* **26**, 215-222 (1977).

S6 Pereira, E., Fiallo, M. M., Garnier-Suillerot, A., Kiss, T. & Kozłowski, H. Impact of aluminium ions on adriamycin-type ligands. *J. Chem. Soc., Dalton Trans.*, 455-459 (1993).
